# Toward a Hybrid Biosensor System for Analysis of Organic and Volatile Fatty Acids in Fermentation Processes

**DOI:** 10.3389/fchem.2018.00284

**Published:** 2018-07-17

**Authors:** Désirée L. Röhlen, Johanna Pilas, Markus Dahmen, Michael Keusgen, Thorsten Selmer, Michael J. Schöning

**Affiliations:** ^1^Institute of Nano- and Biotechnologies, FH Aachen, Jülich, Germany; ^2^Institute of Pharmaceutical Chemistry, Philipps-Universität Marburg, Marburg, Germany; ^3^Institute NOWUM-Energy, FH Aachen, Jülich, Germany; ^4^Institute of Complex Systems 8, Forschungszentrum Jülich, Jülich, Germany

**Keywords:** multi-analyte biosensor, biogas, electrochemical detection, organic acids, volatile fatty acids

## Abstract

Monitoring of organic acids (OA) and volatile fatty acids (VFA) is crucial for the control of anaerobic digestion. In case of unstable process conditions, an accumulation of these intermediates occurs. In the present work, two different enzyme-based biosensor arrays are combined and presented for facile electrochemical determination of several process-relevant analytes. Each biosensor utilizes a platinum sensor chip (14 × 14 mm^2^) with five individual working electrodes. The OA biosensor enables simultaneous measurement of ethanol, formate, d- and l-lactate, based on a bi-enzymatic detection principle. The second VFA biosensor provides an amperometric platform for quantification of acetate and propionate, mediated by oxidation of hydrogen peroxide. The cross-sensitivity of both biosensors toward potential interferents, typically present in fermentation samples, was investigated. The potential for practical application in complex media was successfully demonstrated in spiked sludge samples collected from three different biogas plants. Thereby, the results obtained by both of the biosensors were in good agreement to the applied reference measurements by photometry and gas chromatography, respectively. The proposed hybrid biosensor system was also used for long-term monitoring of a lab-scale biogas reactor (0.01 m^3^) for a period of 2 months. In combination with typically monitored parameters, such as gas quality, pH and FOS/TAC (volatile organic acids/total anorganic carbonate), the amperometric measurements of OA and VFA concentration could enhance the understanding of ongoing fermentation processes.

## 1. Introduction

In light of the depletion of fossil fuels, the public interest of biogas production from renewable resources is steadily increasing. A particular advantage of anaerobic digestion is the ability for simultaneous utilization of industrial waste and thus, providing a promising approach for dealing with another problem of today's world (Angelidaki and Ellegaard, 2003; Komemoto et al., 2009). However, in order to realize the potential of the growing market, several technological and economic aspects need to be improved to ensure process stability and efficient methane (CH_4_) production. Some of these important factors comprise appropriate biogas purification technologies, a suitable feedstock composition and ideal conditions inside the biogas reactor (Weiland, 2010; Andriani et al., 2014; Achinas et al., 2017). The latter is guaranteed by continuous monitoring of various physical and biochemical parameters indicating system stability [pH, alkalinity, gas quality, FOS/TAC (volatile organic acids/total anorganic carbonate)]. Process imbalances are thereby reflected by acidification of the reactor due to accumulation of volatile fatty acids (e.g., acetate, propionate, butyrate) and organic acids, like lactate, formate and alcohols (Nielsen et al., 2007; Boe et al., 2010; Li et al., 2014; Montag and Schink, 2016). Hitherto, estimation of the acid composition is conventionally carried out by gas chromatography (Diamantis et al., 2006), spectroscopy (Falk et al., 2015; Stockl and Lichti, 2018) or HPLC (high-performance liquid chromatography) (Zumbusch et al., 1994; Schiffels et al., 2011). Common disadvantages of these methods are elaborate sample pre-treatment and high costs per analysis, since these are usually executed by external service laboratories. Obviously, the main drawback is the inevitable time delay between sampling and availability of the results, making immediate intervention impossible and therefore represent an element of uncertainty for the plant operators. For these reasons, the acid content is typically only analyzed once or twice per month. In order to overcome this problem, biosensors have been developed as reliable tools for fast and accurate analysis of several compounds.

Much attention has been paid to the development of lactate and ethanol biosensors, due to their diverse applications in food industry and healthcare (Goriushkina et al., 2009; Rathee et al., 2016). Apart from that, several studies imply an association between these intermediates and process stability of the biogas reactor (Pipyn and Verstraete, 1981; Crable et al., 2011). For this reason, the development and optimization of an organic acid (OA) biosensor, comprising enzymes for the specific detection of d/l-lactate, formate and ethanol was a subject of earlier studies (Pilas et al., 2017). In contrast, only a limited number of volatile fatty acid (VFA) biosensors have been described in the literature up to now. The detection of these analytes was accomplished with microbial fuel cells (Kaur et al., 2013), microbial electrolysis cells (Jin et al., 2017) or dissovled oxygen probes with an immobilized biofilm (Sweeney et al., 2018). On-line shock sensors, based on microbial fuel cells were also reported in the literature (Schievano et al., 2018). Specific determination of individual substrates, e.g., propionate and acetate, was realized using enzyme-based sensors (Mizutani et al., 2001; Mieliauskiene et al., 2006; Sode et al., 2008).

**Figure 1:**
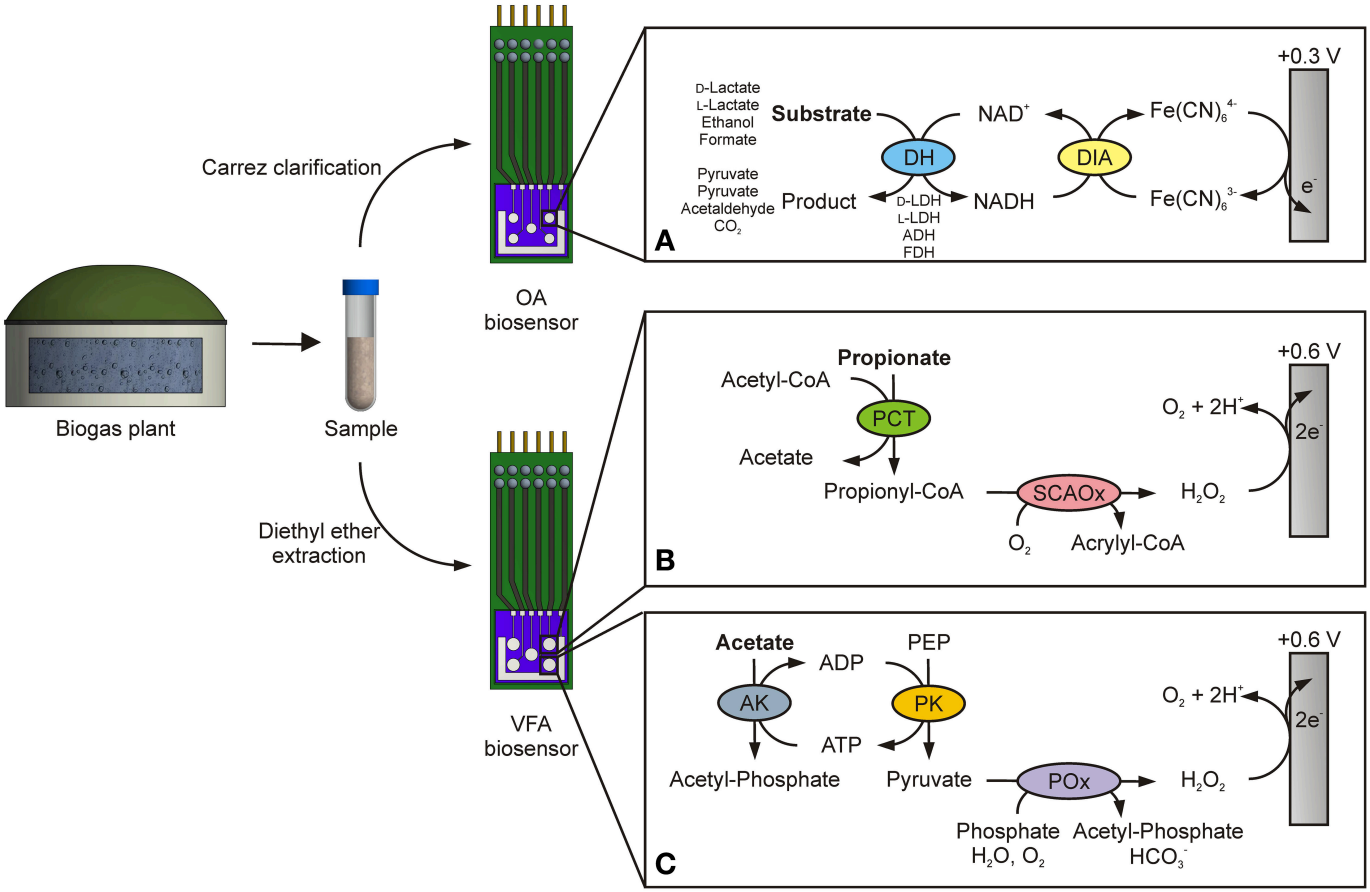
Amperometric detection principles of the organic acid (OA) and volatile fatty acid (VFA) biosensor. A sample of fermentation broth is taken from a biogas plant and pretreated by either Carrez clarification or ether extraction. **(A)** Determination of ethanol, formate, d- and l-lactate is then realized by using a bi-enzymatic system, consisting of a specific dehydrogenase (DH) and diaphorase (DIA). For simultaneous detection, on each working electrode DIA with a different DH is immobilized, namely d-lactate dehydrogenase (d-LDH), l-lactate dehydrogenase (l-LDH), alcohol dehydrogenase (ADH) and formate dehydrogenase (FDH), respectively. The product Fe(CN)_6_^4−^ is re-oxidized at an applied potential of +0.3 V vs. Ag/AgCl. The oxidation of H_2_O_2_ at a working potential of +0.6 V vs. Ag/AgCl is used for measurement of propionate **(B)** and acetate **(C)**. For propionate quantification, propionate CoA-transferase (PCT) and short-chain acyl-CoA oxidase (SCAOx) are used. Combination of acetate kinase (AK), pyruvate kinase (PK) and pyruvate oxidase (POx) enable amperometric detection of acetate.

In the presented approach, the above mentioned OA biosensor is combined with a new established system for concurrent detection of acetate and propionate. Figure 1A shows the enzymatic principle of the OA biosensor for parallel determination of ethanol, formate, d- and l-lactate. In each case, a specific dehydrogenase (DH) is used, which oxidizes its corresponding substrate to acetaldehyde, CO_2_ and pyruvate, respectively. In these reactions, reduction of the cofactor NAD^+^ to NADH is catalyzed. Then, diaphorase (DIA) regenerates the released NADH by reducing the electron acceptor Fe(CN)_6_^3−^ to Fe(CN)_6_^4−^. At an applied working potential of +0.3 V vs. Ag/AgCl, Fe(CN)_6_^4−^ is re-oxidized at the platinum working electrode and the generated current is proportional over a certain linear range to the particular substrate concentration. This method facilitates integration of several analyte-sensing electrodes within one biosensor array. On the contrary, the VFA biosensor works with a different principle for amperometric quantification of acetate and propionate. The working potential is set to +0.6 V vs. Ag/AgCl for anodic oxidation of H_2_O_2_. This compound is produced in both enzymatic reactions. For this reason, propionate CoA-transferase (PCT) and short-chain acyl-CoA oxidase (SCAOx) are immobilized on the working electrode for electrochemical sensing of propionate (Figure 1B). As illustrated in Figure 1C, acetate is indirectly determined by application of acetate kinase (AK), pyruvate kinase (PK) and pyruvate oxidase (POx).

In this work, we present for the first time a modular system for the combined amperometric detection of the OA formate, d/l-lactate and ethanol and for the VFA acetate and propionate. Each biosensor utilizes a different enzyme-based detection principle and thereby, simultaneous determination of four and two analytes was realized. The cross-sensitivity and sensor performance in spiked samples of fermentation broth were investigated. Practical application of both biosensors was demonstrated by long-term monitoring of the OA and VFA concentration in a lab-scale biogas reactor. The proposed hybrid biosensor system proved to be a promising device for rapid and facile quantification of several OA and VFAs in real samples. In this regard, the combination of various parameters enables an enhanced understanding of the process conditions within a biogas reactor and thus facilitates an efficient CH_4_ production.

## 2. Material and methods

### 2.1. Chemicals and reagents

For construction of the biosensors and realization of photometric assays, the following enzymes were used: Acetate kinase from *Escherichia coli* (AK, 150 U mg^−1^), alcohol dehydrogenase from *Saccharomyces cerevisiae* (ADH, 310 U mg^−1^), citrate synthase from porcine heart (CS, 100 U mg^−1^), diaphorase from *Clostridium kluyveri* (DIA, 51 U mg^−1^), formate dehydrogenase from *Candida boidinii* (FDH, 0.49 U mg^−1^), d-lactate dehydrogenase from *Lactobacillus leichmanii* (d-LDH, 213 U mg^−1^), l-lactate dehydrogenase from *Bacillus stearothermophilus* (l-LDH, 174.5 U mg^−1^) and pyruvate kinase from rabbit muscle (PK, 1,000 U mg^−1^) were each obtained from Sigma-Aldrich (St. Louis, MO, USA). Peroxidase from horseradish (HRP, 200 U mg^−1^) and pyruvate oxidase from *Aerococcus viridans* (POx, 25 U mg^−1^) were from Merck (Darmstadt, Germany).

Adenosine 5′-diphosphate sodium salt (ADP), bovine serum albumin (BSA), butyryl coenzyme A lithium salt, capronic acid, ethanol, ethylenediaminetetraacetic acid tetrasodium salt dihydrate (EDTA), flavin adenine dinucleotide disodium salt (FAD), glutaraldehyde solution (GA) (25% in H_2_O), glycerol, sodium d-lactate, potassium ferricyanide (K_3_[Fe(CN)_6_]), potassium ferrocyanide (K_4_[Fe(CN)_6_].3H_2_O), propionyl coenzyme A lithium salt, sodium propionate, sodium pyruvate, thiamine pyrophosphate (TPP), Triton X-100, valeric acid and ZnCl_2_ were also supplied by Sigma-Aldrich. Adenosine 5′-triphosphate disodium salt (ATP), (2,2′-azino-bis(3-ethylbenzothiazoline-6-sulphonic acid) diammonium salt (ABTS), acetyl coenzyme A trilithium salt, sodium formate, sodium l-lactate, nicotinamide adenine dinucleotide (NAD^+^), oxaloacetic acid and phospho(enol)pyruvic acid monopotassium salt (PEP) were purchased from AppliChem (Darmstadt, Germany). Diethyl ether, 5,5′-dithiobis-(2-nitrobenzoic acid), potassium phosphate buffer (K_2_HPO_4_, KH_2_PO_4_), Tris-(hydroxymethyl)-aminomethane, H_2_SO_4_, MgCl_2_ and NaOH were from Carl Roth GmbH & Co. KG (Karlsruhe, Germany). Dithiothreitol (DTT), sodium phosphate buffer (Na_2_HPO_4_, NaH_2_PO_4_), sodium acetate and sodium butyrate were acquired from Merck (Darmstadt, Germany). d-Desthiobiotin was provided by IBA (Göttingen, Germany).

### 2.2. Cloning

The propionate sensing system is composed of two recombinantly produced enzymes, a propionate CoA-transferase (PCT, EC 2.8.3.1) from *Clostridium propionicum* and a short-chain acyl-CoA oxidase (SCAOx, EC 1.3.3.6) derived from *Arabidopsis thaliana*. Fabrication of the propionate electrode involved cloning of the corresponding genes into designated expression vectors, biomass production and purification of the proteins.

Based on the published sequence (Hayashi et al., 1999), the SCAOx gene was codon-optimized for expression in *E. coli* and synthesized by Eurofins Genomics (Ebersberg, Germany). Moreover, internal restriction sites for *Esp*3I were removed. The resulting sequence was amplified by polymerase chain reaction (PCR) using two primers (SCAOx for 5′-AAGCTCTTCAATGGCGGTTCTGTCAAGCG-3′ and SCAOx rev 5′-AAGCTCTTCACCCTTACAAACGAGAGCGGGTAGC-3′) with incorporated *Lgu*I restriction sites (underlined). After analysis of the purified PCR product by chip electrophoresis (MCE-202 MultiNA; Shimadzu, Duisburg, Germany), the SCOAx gene was digested with *Lgu*I and cloned into a pENTRY vector (IBA, Göttingen, Germany). *E. coli* DH5α-competent cells were transformed with the resultant plasmid. Following sequence analysis (Eurofins Genomics, Ebersberg, Germany), the SCAOx gene cassette was subcloned into the *Esp*3I site of a StarGate Acceptor Vector (IBA, Göttingen, Germany), containing an N-terminal-fused Strep-tag. Expression plasmids harboring propionate CoA-transferase fused to an N-terminal Strep-tag, were synthesized as previously described (Bijtenhoorn, 2005).

### 2.3. Gene expression and protein purification

*E. coli* BL21(DE3) cells carrying the constructed plasmids were used for production of the recombinant proteins. Following pre-cultivation at 28°C for approx. 15 h in 100 mL LB medium (Luria-Bertani) with 50 μg/mL carbenicillin, the culture was inoculated to 500 mL of the same medium. At optical density (OD_578*nm*_) of 0.6–0.8, gene expression was initiated by treatment with 200 ng/ml AHT. Post induction, cells harboring recombinant SCAOx were incubated for 2 h at 28°C and finally harvested by centrifugation. Cell pellets were washed once with 50 mL PBS [137 mM NaCl, 2.7 mM KCl, 10 mM Na_2_HPO_4_, 1.8 mM KH_2_PO_4_ (pH 7.4)] and afterwards stored at −80°C until used for protein purification. PCT-containing *E. coli* cells were cultivated for 3 h at 28°C post induction and washed with 50 mL TBS [50 mM Tris, 150 mM NaCl (pH 7.5)] prior storage.

Purification of SCAOx was accomplished by affinity chromatography with a Strep-Tactin Macroprep column (IBA GmbH, Göttingen, Germany) as outlined earlier (Röhlen et al., 2017). Briefly, cell pellets were suspended in 100 mM sodium phosphate buffer (pH 7.5, supplemented with 150 mM NaCl and 10 μM FAD) and lysed by sonication. Next, cell debris was pelleted by ultracentrifugation and the clear supernatant was loaded onto the equilibrated column. Elution of the protein was effected by addition of 2.5 mM d-desthiobiotin in aforementioned buffer. Protein concentration and purity were verified by sodium dodecyl sulfate polyacrylamide gel electrophoresis (SDS-PAGE) and Bradford analysis. Purified protein fractions were concentrated by ultrafiltration and 10 vol% glycerol was added for storage at −20°C. Similarly, recombinant PCT was purified using 100 mM Tris-HCl pH 8.0 (supplemented with 150 mM NaCl, 1.0 mM EDTA, 1.0 mM DTT) as resuspension buffer and additionally 2.5 mM d-desthiobiotin for subsequent elution. Prior enzyme immobilization, the storage buffer was exchanged with 100 mM sodium phosphate buffer pH 7.5, 1.0 mM DTT, 1.0 mM EDTA.

### 2.4. Enzyme activity measurements

Enzyme activities were determined spectrophotometrically at 25°C in 1 mL reaction mixture using an Ultrospec 2100 pro spectrophotometer (Amersham Biosciences, UK).

Short-chain acyl-CoA oxidase activity was measured in a coupled assay with HRP (Baltazar et al., 1999). The assay mixture included 100 mM sodium phosphate buffer (pH 7.4), supplemented with 0.05 mM FAD, 0.05 mM acyl-CoA, 2.0 mM ABTS and 5.0 U HRP. The reaction was started by addition of the enzyme and the increase in absorbance at 405 nm, due to oxidation of ABTS, was monitored. A molar extinction coefficient ϵ_405nm_ of 18.4 mM^−1^ cm^−1^ was used for calculation of enzyme activities (Werner et al., 1970).

Propionate CoA-transferase activity was determined by detection of free CoA via a coupled citrate synthase-DTNB reaction (Selmer et al., 2002). The reaction mixture consisted of 100 mM sodium phosphate (pH 7.4), 0.05 mM propionyl-CoA, 20 mM sodium acetate, 1 mM DTNB (5,5′-dithiobis-(2-nitrobenzoic acid)) and 1 mM oxaloacetate and 3 U citrate synthase. The assay was initiated by addition of PCT and the change in absorbance was followed at 415 nm. Enzyme activities were calculated using a molar extinction coefficient ϵ_415nm_ of 14.14 mM^−1^ cm^−1^.

### 2.5. Biosensor preparation

The multi-parameter biosensor chips (14 × 14 mm^2^) were fabricated by thin-film technology (Pilas et al., 2018). Each biosensor array consists of five individual platinum working electrodes and an additional counter electrode (area 40.5 mm^2^). The diameter of each working electrode of the OA biosensor was 2 mm, whereas the working electrodes of the VFA biosensor were slightly larger (∅ 2.5 mm) for immobilization of an increased volume of enzyme solution. Before the enzymes were immobilized onto the electrodes, the biosensor chips were cleaned by electrochemical treatment in 0.5 M H_2_SO_4_ until a stable signal was obtained (+2.0 V vs. Ag/AgCl for 2 min and subsequent cyclic voltammetry from −0.2 to +1.4 V vs. Ag/AgCl).

Enzymes were immobilized by chemical cross-linking with 0.4 vol% GA solution, supplemented with 10 vol% glycerol and 2% BSA. In case of the OA biosensor, each working electrode was endowed with a different DH (ADH, FDH, d-LDH and l-LDH, respectively) in combination with the DIA. Thereby, a volume of 1.5 μL of each enzyme mixture was applied on one of the working electrodes. The fifth working electrode served as a reference and was modified only with the inert protein BSA, which does not exhibit any catalytic activity. Details of exact enzyme loadings on the OA biosensor were given earlier (Pilas et al., 2017).

For construction of the VFA biosensor, GA concentrations were adjusted to 0.24 vol% (propionate electrode) and 0.7 vol% (acetate electrode) each with 2% BSA. The propionate-specific electrode contained 0.032 U PCT and 0.057 U SCAOx embedded in the BSA-GA matrix. Acetate detection was accomplished by an enzyme layer consisting of 3 U POx, 6 U PK and 6 U AK. Each electrode was equipped with 3 μL of the corresponding enzyme mixture.

### 2.6. Experimental set-up and operation

**Figure 2:**
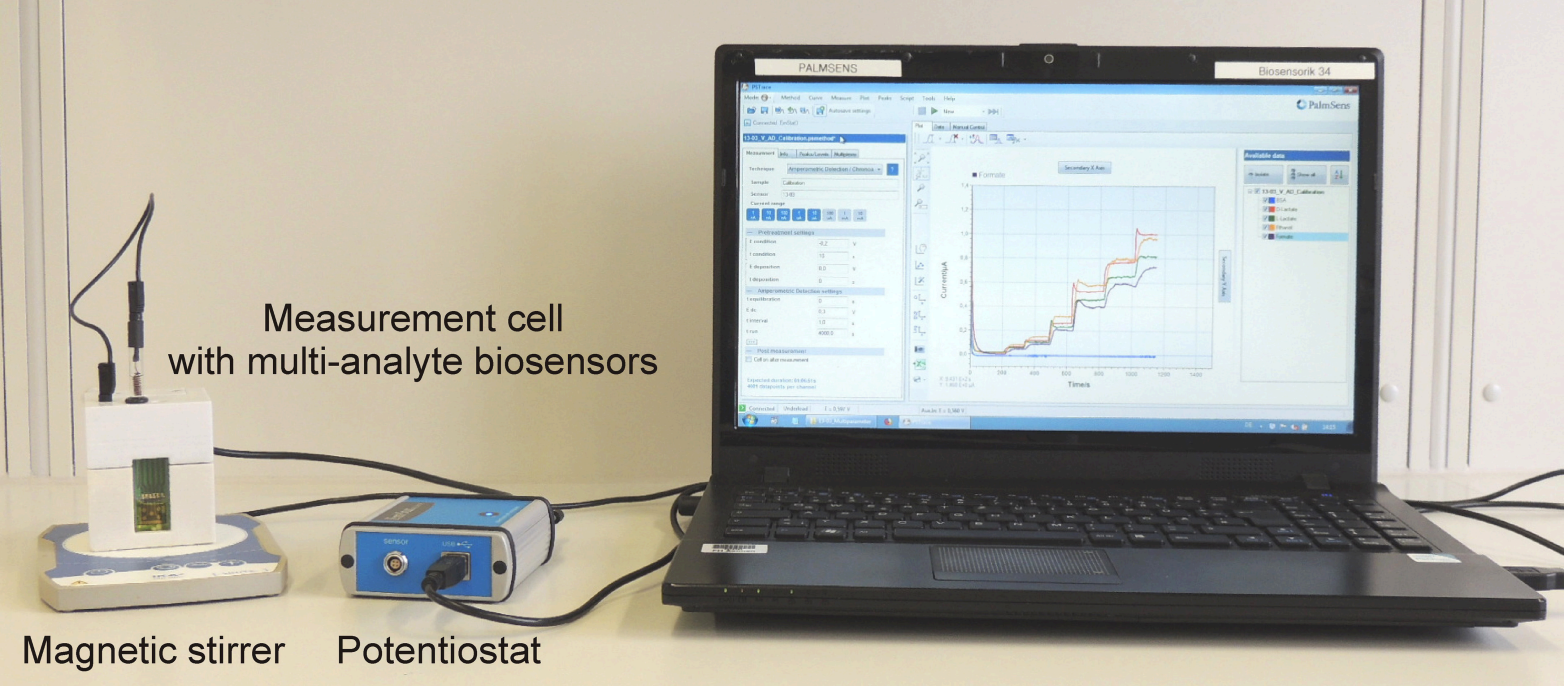
Photograph of measurement set-up with biosensor and Ag/AgCl reference electrode connected to a potentiostat and software device.

All electrochemical experiments were conducted at room temperature in a three-electrode arrangement with a Ag/AgCl reference electrode (with KCl as inner electrolyte; Sensolytics, Bochum, Germany) and the biosensor, comprising the working electrode (each with five working electrodes per biosensor) and counter electrode. The set-up consisted of a custom-made measurement cell connected to a potentiostat with integrated multiplexer (EmStat3 and MUX16, PalmSens BV, Houten, Netherlands) (Pilas et al., 2018). Figure 2 shows an image of the applied set-up. For operation of the OA biosensor, a working potential of +0.3 V vs. Ag/AgCl was applied for anodic oxidation of enzymatically produced Fe[CN]_6_^4−^. Standard reaction mixture contained 2.5 mM NAD^+^ and 2 mM Fe(CN)_6_^3−^ dissolved in 100 mM potassium phosphate buffer (pH 7.5).

Detection of acetate and propionate with the VFA biosensor was realized at an applied potential of +0.6 V vs. Ag/AgCl for oxidizing H_2_O_2_. Measurements were carried out in 100 mM potassium phosphate buffer (pH 7.0), supplemented with 5 mM MgCl_2_, 0.6 mM TPP, 0.02 mM FAD, 2 mM PEP, 1 mM ATP and 0.4 mM acetyl-CoA. Due to the rapid loss of enzyme activity, an additional 11.84 U PK and 2.5 U AK were added to the reaction mixture. For both biosensors, a volume of 2 mL of the corresponding measurement solution was used. Homogeneous distribution of the calibration and sample solution, respectively, was accomplished with a magnetic stirrer.

### 2.7. Analysis of fermentation broth from biogas plants

Samples of fermentation broth (approximately 50 mL) were collected one-time from three industrial continuously operated biogas plants in Germany and regularly from a lab-scale biogas reactor. Fermentation sludge from the industrial plants was stored frozen at −21°C until further measurements, whereas samples from biogas test reactor were analyzed immediately after sampling. For application of the OA and VFA biosensor, as well as analysis by reference techniques, samples were pretreated by two different procedures (see Figure 1). On the one hand, samples for the OA biosensor were clarified by Carrez precipitation (Carrez, 1908). A volume of 10 mL of fermentation sludge was mixed carefully with 2 mL of 0.68 M K_4_[Fe(CN)_6_]·3H_2_O and subsequently, 2 mL of 2 M ZnCl_2_ were added and agitated again. Following, precipitation was induced by addition of 5 mL of 0.4 M NaOH and the final volume was adjusted to 20 mL with deionized water. Insoluble compounds were then separated by centrifugation and the clear supernatant was used for further investigations. For comparative studies, the concentration of ethanol, formate, d- and l-lactate was as well determined with commercial photometric kits (Megazyme International, Wicklow, Ireland) following the manufacturers′ instructions. On the other hand, a diethyl ether extraction method was adopted for analysis of acetate and propionate by the VFA biosensor (Schiffels et al., 2011). Therefore, 300 μL of the fermentation broth were mixed with 0.2 g NaCl, 50 μL concentrated HCl and 800 μL diethyl ether. Samples were briefly centrifuged and the ether phase was diluted into 600 μL sodium phosphate buffer pH 7.0. The content of VFA was additionally quantified by a gas chromatograph (GC-2010, Shimadzu, Duisburg, Germany) equipped with a poly ethylene glycol column (FS-FFAP-CB-0.25, CS-Chromatographie Service GmbH, Langerwehe, Germany) and a flame ionization detector.

The OA and VFA biosensors were also applied for the long-term monitoring of a lab-scale biogas reactor (CSTR-10S, Bioprocess Control AB, Lund, Sweden) with 0.01 m^3^ working volume, equipped with a wall jacket and an external water bath [ICC basic pro 9, IKA (Staufen, Germany)] for operation at constant temperature (40°C). The continuously operated reactor received a daily feeding of approximately 60 g of sugar cane silage. Analysis of the biogas composition (CH_4_ and CO_2_) was performed online by an infrared sensor system (BlueSens, Herten, Germany) on a daily basis. The pH and FOS/TAC were determined offline once per week. During a period of 2 months, digestate samples (50 mL) were taken once a week, purified as described above and subsequently used for electrochemical analysis.

Prior application of the biosensors in real samples, calibration curves were obtained by monitoring the increase in the current signal after successive addition of a stock solution with defined concentration (each consisting of all analytes). Real samples were analyzed by subsequent titration to the reaction buffer, resulting in different dilutions. Based on the sensitivities of the calibration curves, the concentration of each analyte was calculated for each dilution step.

## 3. Results and discussion

### 3.1. Sensor characteristics

**Figure 3:**
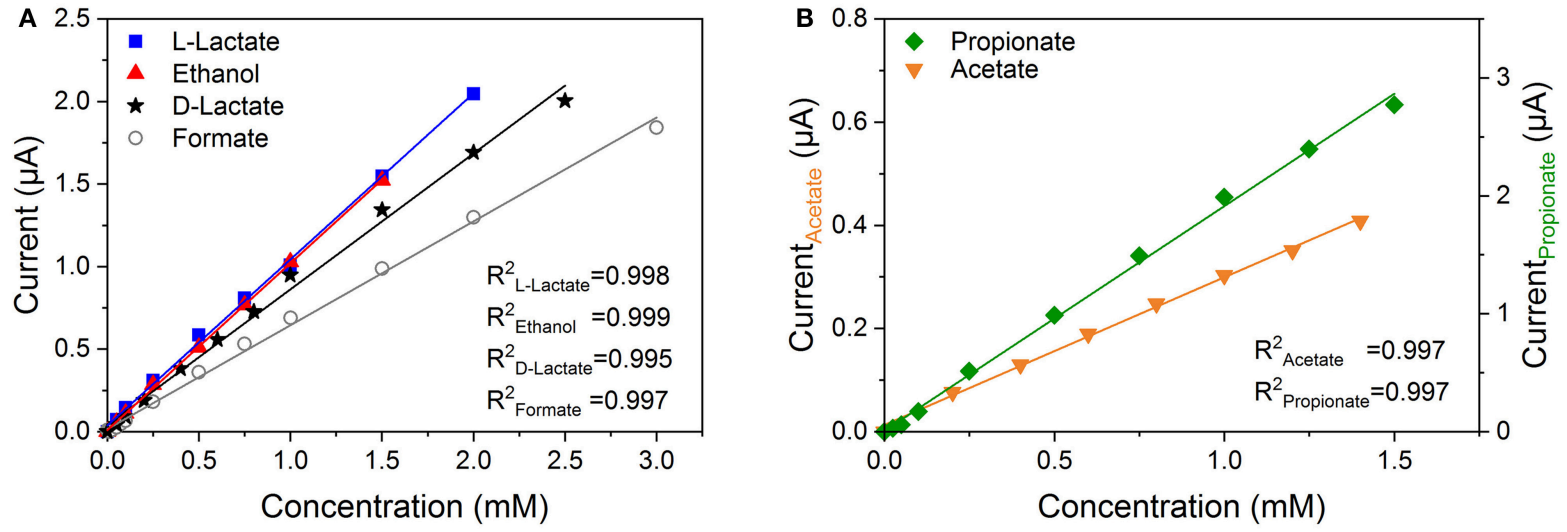
Calibration curves of **(A)** the organic acid and **(B)** the volatile fatty acid biosensor.

The sensor performances were characterized in terms of sensitivity and linear detection range by successive addition of standard solutions with defined concentrations of each analyte. In Figures 3A,B the calibration curves of the OA and VFA biosensor are presented. The individual electrodes exhibited a linear relationship between current increase and analyte concentration. Table 1 summarizes the results obtained for both biosensors. The four analyte sensing elements of the OA biosensor had a similar linear detection range with a sensitivity from 0.64 to 1.16 μA mM^−1^. Substantially, the different electrodes of the VFA biosensor possessed a sensitivity of 0.27 and 2.11 μA mM^−1^ for acetate and propionate, respectively. In literature, a propionate biosensor based on the same detection principle was reported, hereby, the enzymes were immobilized within a polymer of poly(vinyl alcohol) with styrylpyridinium groups (PVA-SbQ) (Sode et al., 2008). This biosensor displayed a linear detection range of 10–100 μM with a sensitivity of 1.7 μA mM^−1^ cm^−2^. With a normalized sensitivity of 42.9 μA mM^−1^ cm^−2^ the propionate electrode of the VFA biosensor shows an almost 25 times higher sensitivity over a broader linear range.

**Table 1:** Sensor characteristics of the volatile fatty acid (VFA) and organic acid (OA) biosensor operated at an applied potential of +0.6 and +0.3 V vs. Ag/AgCl, respectively.

**Biosensor**	**Analyte**	**Sensitivity**	**Linear range**
		**(μA mM^−1^)**	**(mM)**
VFA	Propionate	2.11 ± 0.41	0–1.5
VFA	Acetate	0.27 ± 0.05	0–1.4
OA	d-Lactate	0.89 ± 0.03	0–2.5
OA	l-Lactate	1.16 ± 0.06	0–2.0
OA	Formate	0.64 ± 0.02	0–3.0
OA	Ethanol	1.12 ± 0.03	0–1.5

Only few electrochemical enzyme-based acetate biosensors are mentioned in literature. One of them was also a tri-enzyme system, consisting of AK, PK and POx, which were entrapped on the platinum electrode in a membrane of polydimethylsiloxane (PDMS) (Mizutani et al., 2001). Measurements were performed at an applied potential of −0.4 V vs. Ag/AgCl for monitoring of the oxygen consumption. Under this condition, a linear correlation between current signal and acetate concentration was obtained in a narrow range of 5 μM to 0.5 mM compared to the VFA biosensor of the present study (0.2–1.4 mM).

### 3.2. Evaluation of interferences

Given the complex chemical composition of biogas sludges, interfering effects of different substances were investigated prior to application of the sensors in real samples. The examined compounds were selected according to the substrate spectrum of the employed enzymes and potential occurrence in the fermentation broth. In each measurement, the respective compound was added individually to the reaction buffer and substrate-related current changes were determined. All tests were conducted in triplicate. Table 2 summarizes the results of the influence of potential interferents on the sensor response of both biosensors. Obtained current responses were normalized against current signals monitored for the intended substrate.

**Table 2:** Effect of potential interferents on the different electrodes of the volatile fatty acid (VFA) and organic acid (OA) biosensor.

	**VFA biosensor**		**OA biosensor**				
**Interferent**	**Acetate**	**Propionate**	**d-Lactate**	**l-Lactate**	**Formate**	**Ethanol**	**BSA Blank**
	**(%)**	**(%)**	**(%)**	**(%)**	**(%)**	**(%)**	**(%)**
Acetate	100	0	–	–	0	0	0
Propionate	6	100	–	–	0	0	0
d-Lactate	–	–	100	0	0	0	0
l-Lactate	–	–	0	100	0	0	0
Formate	3	–	0	0	100	0	0
Ethanol	0	–	0	0	0	100	0
Pyruvate	117	–	0	0	0	0	0
Malate	–	–	9	12	4	7	0
Butyrate	0	28	–	–	0	–	0
Valerate	–	1	–	–	–	–	0
Capronate	–	0	–	–	–	–	0
Glycerol	0	–	–	–	–	–	0
n-Propanol	–	–	–	–	–	61	0
n-Butanol	–	–	–	–	–	43	0
Methanol	–	–	–	–	0	0	0
Ascorbate	–	–	75	53	–	–	100
Cysteine	–	–	36	29	–	–	65
Urea	–	–	1	1	–	–	6

*Change in the current signal was normalized to the current obtained for the intended substrate (−, not evaluated)*.

The selectivity of the VFA biosensor was investigated by introduction of 0.5 mM substrate to the reaction mixture. Several different short-chain fatty acids were deployed for cross-sensitivity tests with the propionate-sensing electrode. Relevant current changes were solely observed for butyrate (28%), a natural substrate for PCT (Selmer et al., 2002) and, in the activated form (butyryl-CoA), for SCAOx (Hayashi et al., 1999). However, the combination of both enzymes strongly favors the enzymatic conversion of propionate (100%), which is consistent with data from the literature describing a propionate electrode and a photometric assay based on the same enzyme cascade (Rajashekhara et al., 2006; Sode et al., 2008).

The concentration and specific ratio of volatile fatty acids in a biogas reactor is highly dependent on the feedstock and type of digestion. Although butyric acid is usually present in the biogas broth, and thus both substrates compete for the same catalytic binding site of the PCT, typical concentrations of this fatty acid are decisively lower compared to propionate (Franke-Whittle et al., 2014). Therefore, the usual ratio of the acids on the one hand and the affinity of the biosensor for the specific substrates on the other hand favor the detection of propionate in the fermentation broth. In addition, due to the thermodynamic unfavorable conditions for propionate degradation, the short-chain fatty acid persists longer in the fermentation broth than other volatile fatty acids and is therefore regarded as a reliable indicator for process monitoring (Nielsen et al., 2007).

The acetate-sensing electrode was also subjected to interference study using potential AK substrates (propionate, formate, ethanol, butyrate, and glycerol) and pyruvate, the main substrate of POx. While no signal response was observed with ethanol, butyrate and glycerol, slight current increase was monitored for propionate (6%) and formate (3%). Interference with propionic acid was likewise reported by different acetate biosensors using AK (Tang and Johansson, 1997; Mizutani et al., 2001). Nevertheless, our findings suggest a clear preference of AK for acetate over the other substrates tested. Apart from this, both substrates are naturally not present to the same extent and the propionate concentration is significantly lower as compared to acetate (Montag and Schink, 2016). Cross-sensitivity with propionate thereby has rather little effect on the amperometric acetate detection. Not surprisingly, the acetate sensor showed the highest sensor response upon addition of pyruvate (117%). However, as intermediate of several metabolic pathways (Zhou et al., 2018), pyruvate degrades rapidly and thus extracellular concentrations of the POx substrate are negligible compared to the acetate levels in the biogas medium. Previous studies on the accumulation of extracellular metabolites from *E. coli* under anaerobic conditions showed only minimal levels of pyruvic acid compared to the concentration of acetate and other acids (Kim et al., 2015; Yasid et al., 2016).

Evaluation of possible susceptibility of the OA biosensor to potential interferents was accomplished by observing the change in current signal after addition of several substrates (each 1 mM) to the measurement solution. Both lactate electrodes exhibited sensitivity toward ascorbate, cysteine and to some small extent to urea. All of these compounds are known reducing agents at the applied positive working potentials (Sprules et al., 1995; Palmisano et al., 2000). For this reason, an increase in the current signal was observed for the electrode covered with BSA. The ethanol electrode was also sensitive to other alcohols, namely n-propanol (61%) and n-butanol (43%). This interference is mainly caused by the broad substrate spectrum of the applied ADH from *S. cerevisiae* (Plapp et al., 1993). The substrate specificity of electrochemical ethanol biosensors is generally a great challenge, since detection principles based on the enzyme alcohol oxidase show this characteristic behavior, too (Azevedo et al., 2005).

**Figure 4:**
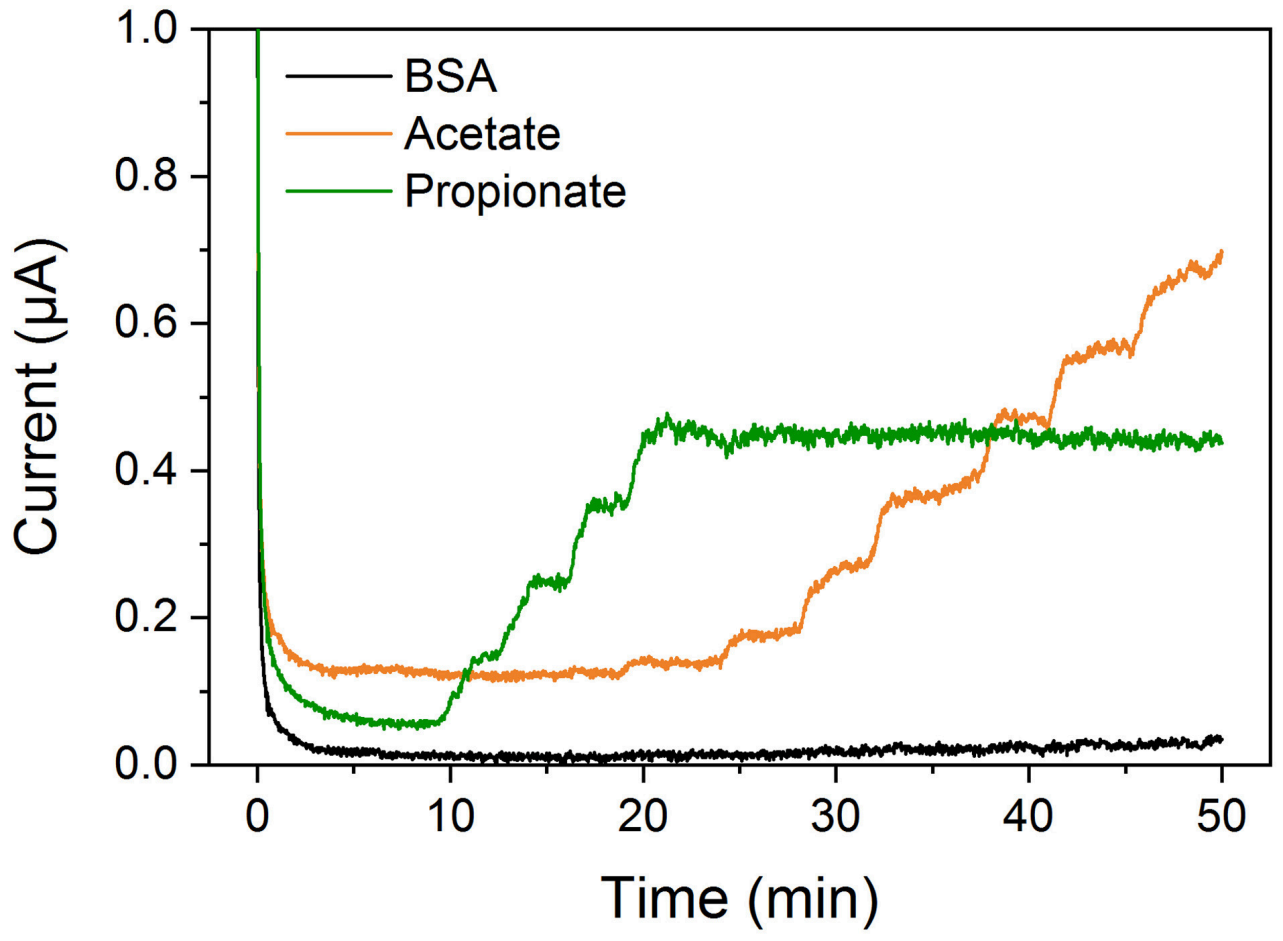
Chronoamperometric measurement with the VFA biosensor in 100 mM potassium phosphate buffer (pH 7.0) with 5 mM MgCl_2_, 0.6 mM TPP, 0.02 mM FAD, 2 mM PEP, 1 mM ATP and 0.4 mM acetyl-CoA, 11.8 U PK and 2.5 U AK. Cross-talk free behavior is demonstrated by subsequent addition of propionate and acetate, respectively.

Due to the substrate range of AK on the one hand and the PCT-catalyzed formation of acetate from acetyl-CoA on the other hand, potential cross-talk between the two VFA electrodes was investigated by successive addition of the analytes. As depicted in Figure 4, only the corresponding electrode showed a current response upon introduction of the substrate. Similarly, no inadvertent interactions were observed for simultaneous determination of ethanol, formate, d- and l-lactate with the OA biosensor as described earlier (Pilas et al., 2017).

### 3.3. Evaluation of sensor performance in spiked samples

**Figure 5:**
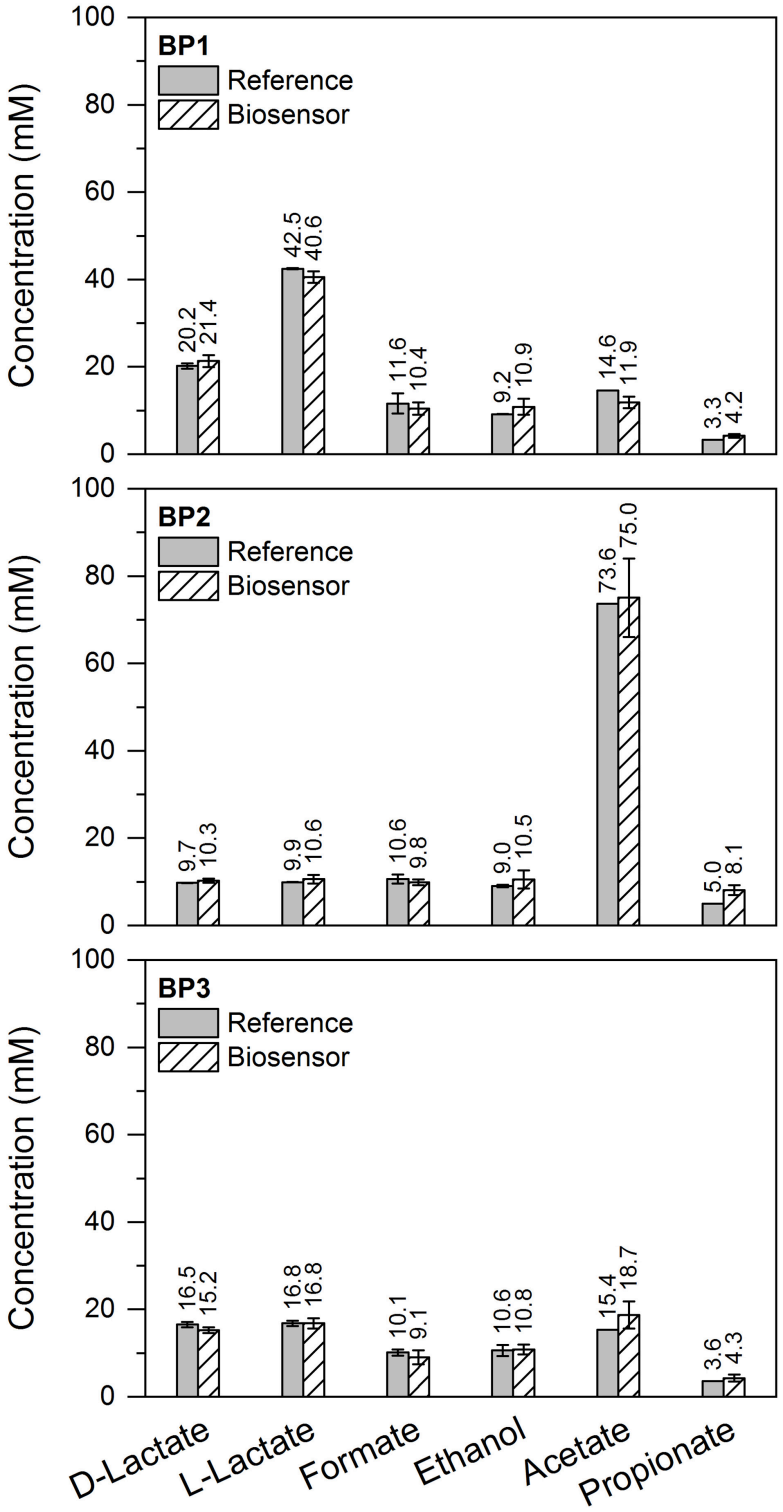
Comparison between the results obtained by the two biosensors and the corresponding reference technique. Samples were collected from three different biogas plants (BP1, BP2, BP3) and spiked with 20 mM acetate, 5 mM propionate and 10 mM ethanol, formate, d- and l-lactate. For detection of ethanol, formate, d- and l-lactate a photometric reference technique was applied, and acetate and propionate were additionally quantified by gas chromatography.

For evaluation of the sensor performance in real samples and complex matrices, sludge samples from three different biogas plants (BP1 to BP3) were collected. Biogas production in BP1 was achieved by mono-digestion of maize silage, whereas in BP2 additionally cattle slurry was applied. The feedstock of BP3 consisted of maize silage, cattle slurry and manure. The type of feedstock used for anaerobic digestion mainly influences the viscosity of the fermentation broth. In order to test the biosensors in various media compositions, biogas plants with different feedstocks were selected. After sampling, fermentation sludges were spiked with 20 mM acetate, 5 mM propionate and each 10 mM ethanol, formate, d- and l-lactate, respectively. The concentration of VFA and OA was determined with the two biosensors and for comparative studies by gas chromatography and commercial photometric kits. Figure 5 provides a comparison of the results obtained by the biosensors and reference techniques. For all three samples, the amperometrically determined concentrations correlate well with the corresponding conventional method. These findings demonstrate successfully the potential of simultaneous and rapid monitoring of several analytes in complex media by application of the electrochemical hybrid biosensor system.

### 3.4. Monitoring of a lab-scale biogas reactor

The formation of biogas from organic matter is a complex procedure carried out by a consortium of different microorganisms. It involves four phases: hydrolysis, acidogenesis, acetogenesis and methanogenesis. In the first step, complex polymers, like carbohydrates, fats and proteins are degraded into smaller molecules. Hydrolysis is followed by the acid-forming step, the acidogenesis. At this stage, the fermenting bacteria produce volatile fatty acids, alcohols as well as H_2_, CO_2_, and NH_4_. Then, acetogenic and syntrophic bacteria metabolize fatty acids and alcohols into acetate, H_2_ and CO_2_. Finally, acetate and hydrogen are used by methanogenic archaea to produce CH_4_ and CO_2_. In an anaerobic digester, these four processes occur concurrently. In order to successfully maintain the biogas production, suitable detection systems for specific key parameters are required. Therefore, the developed hybrid biosensor system was applied for the long-term monitoring of a lab-scale biogas reactor (0.01 m^3^), operated at mesophilic conditions (40°C) with sugar cane silage as feedstock. Besides the concentration of OA and VFA, also several physical- and chemical parameters were investigated for a period of 2 months.

**Figure 6:**
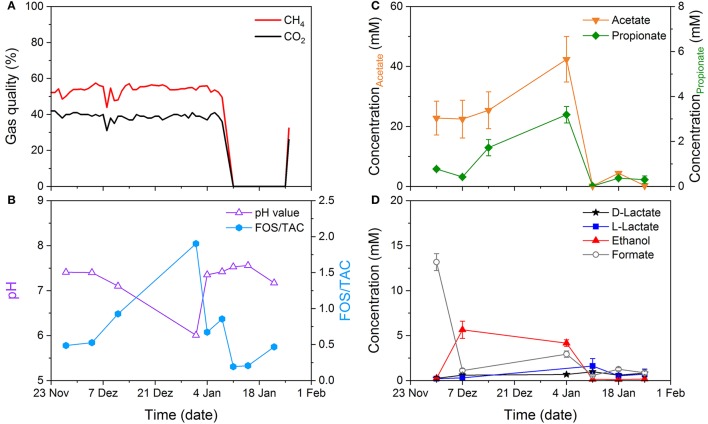
Reactor performance of a lab-scale biogas reactor. **(A)** Online monitoring of gas quality (CH_4_ and CO_2_). **(B)** pH and FOS/TAC were analyzed offline. Concentration of **(C)** volatile fatty acids (acetate and propionate), and **(D)** organic acids (ethanol, formate, d- and l-lactate) measured with the VFA and OA biosensor, respectively.

Figure 6 provides an overview of all the data obtained in this time frame. The content of CH_4_ and CO_2_ in the produced biogas was analyzed online. Basically, the biogas composition was stable during the first 44 days with 53.7 ± 2.7% and 39.0 ± 7.8% of CH_4_ and CO_2_, respectively (Figure 6A). These quantities represent typical values reported in literature (Herout et al., 2011). Between day 45 and 47 the fermenter was temporarily shut down for technical reasons, which is reflected in the sudden loss of CH_4_ and CO_2_ release. Biogas production was resumed 5 days after restart of the digester. In addition to end-product determination, the ratio of volatile fatty acid to total alkalinity (FOS/TAC) and pH are regularly monitored process parameters and are here depicted in Figure 6B. While the pH optimum is typically defined between 6.5 and 7.5, opinions vary regarding optimum VFA concentrations and thus FOS/TAC levels, but agree on the fact, that normal VFA levels highly depend on the individual system (Angelidaki et al., 1993; Franke-Whittle et al., 2014). Therefore, stable concentrations are considered more substantial than the magnitude (Hamawand and Baillie, 2015). During the observed period, FOS/TAC concentrations fluctuated between 0.5 and 1.0 until fermenter stoppage with one peak at day 36. This sudden increase was accompanied with a drop in pH, caused by acidification of the medium due to VFA accumulation. This change in the acid composition was detectable with the hybrid biosensor, too. As depicted in Figure 6C, acetate and propionate concentrations, which have a decisive impact on FOS/TAC, showed a similar curve progress during the observed time frame. Minor changes in organic acid and alcohol content were detected by the OA sensor. For each measurement point, samples were also analyzed using the conventional techniques as described in section 3.3. Again, our findings were in good agreement with the reference methods (data not shown). The results demonstrate a successful long-term application of the hybrid biosensor system for monitoring of acid composition changes. The detection of essential precursors and intermediates of the anoxic food chain, realized by the OA sensor, is a useful extension to established process parameters, as these are usually not covered by conventional monitoring systems. The combined determination of the different acids leads to an improved understanding of the events that occur during fermentation. Thus, potential bottlenecks of the process can be identified and eliminated immediately.

## 4. Conclusion

Nowadays, monitoring of organic and volatile fatty acids in anaerobic fermentation processes is only feasible by laborious techniques, such as HPLC or GC. The analysis by these methods, however, is time-consuming and results are typically provided with some delay after sampling. In this study, two different enzyme-based biosensors were demonstrated as a hybrid system for amperometric detection of several process-relevant intermediates: on the one hand, an OA biosensor for simultaneous determination of ethanol, formate, d- and l-lactate, and on the other hand, a VFA biosensor for electrochemical quantification of acetate and propionate. The effect of various potential interferents on the sensor signal of both biosensors was investigated and results revealed only limited cross-sensitivity. The acetate electrode showed 6% response to propionate and the propionate-sensing electrode was sensitive to other volatile fatty acids (28 and 1% to butyrate and valerate, respectively). The ethanol sensor displayed sensitivity to other alcohols, such as n-propanol (61%) and n-butanol (43%). Nevertheless, both biosensors showed satisfactory cross-talk behavior and the potential for practical application in complex matrices was demonstrated. These findings were also verified by evaluation of the sensor performance in spiked samples of fermentation broth from different biogas plants. A good correlation was obtained between the biosensors and conventional reference techniques. Additionally, the electrochemical biosensor system was used for the first time for long-term monitoring of the acid composition in a lab-scale biogas reactor. Application of such a device would greatly enhance the overall understanding of complex fermentation processes. In comparison to traditional analytical procedures, the presented hybrid biosensor system offers facile, rapid and on-site determination of multiple acids, due to a portable measurement set-up.

Future work will focus on the development of a common procedure for sample preparation, which is suitable for all analytes and both biosensors. In this regard, usage of the crude extract for the electrochemical measurements is envisaged, so that sample pretreatment is not required at all. Application of such a compact monitoring device for determination of acetate, propionate, ethanol, formate, d- and l-lactate would enable early detection of imbalances in anaerobic fermentation processes. Moreover, the broad substrate spectrum of SCAOx allows a future extension of the system by substitution of PCT with other enzymes providing activated short fatty acids. Therefore, the combination of butyrate-specific enzymes with SCAOx would permit a more precise determination of the VFA content in the biogas reactor.

## Author contributions

DR and JP designed and performed the experiments, analyzed the data and wrote the manuscript. MD contributed samples from different biogas plants. TS, MK, and MS supervised the experiments, critically reviewed and edited the manuscript.

### Conflict of interest statement

The authors declare that the research was conducted in the absence of any commercial or financial relationships that could be construed as a potential conflict of interest.
